# High pH microbial ecosystems in a newly discovered, ephemeral, serpentinizing fluid seep at Yanartaş (Chimera), Turkey

**DOI:** 10.3389/fmicb.2014.00723

**Published:** 2015-01-19

**Authors:** D'Arcy R. Meyer-Dombard, Kristin M. Woycheese, Erin N. Yargıçoğlu, Dawn Cardace, Everett L. Shock, Yasemin Güleçal-Pektas, Mustafa Temel

**Affiliations:** ^1^Department of Earth and Environmental Sciences, University of Illinois at ChicagoChicago, IL, USA; ^2^Department of Geosciences, University of Rhode IslandKingston, RI, USA; ^3^School of Earth and Space Exploration, Arizona State UniversityTempe, AZ, USA; ^4^Department of Chemistry and Biochemistry, Arizona State UniversityTempe, AZ, USA; ^5^Faculty of Science, University of IstanbulIstanbul, Turkey; ^6^Department of Freshwater Biology, Istanbul UniversityIstanbul, Turkey

**Keywords:** deep subsurface, serpentinization, Yanartaş (Chimera) Turkey, Tekirova ophiolite, high pH springs, ultramafic

## Abstract

Gas seeps emanating from Yanartaş (Chimera), Turkey, have been documented for thousands of years. Active serpentinization produces hydrogen and a range of carbon gases that may provide fuel for life. Here we report a newly discovered, ephemeral fluid seep emanating from a small gas vent at Yanartaş. Fluids and biofilms were sampled at the source and points downstream. We describe site conditions, and provide microbiological data in the form of enrichment cultures, Scanning electron microscopy (SEM), carbon and nitrogen isotopic composition of solids, and PCR screens of nitrogen cycle genes. Source fluids are pH 11.95, with a Ca:Mg of ~200, and sediments under the ignited gas seep measure 60°C. Collectively, these data suggest the fluid is the product of active serpentinization at depth. Source sediments are primarily calcite and alteration products (chlorite and montmorillonite). Downstream, biofilms are mixed with montmorillonite. SEM shows biofilms distributed homogeneously with carbonates. Organic carbon accounts for 60% of the total carbon at the source, decreasing downstream to <15% as inorganic carbon precipitates. δ^13^C ratios of the organic carbon fraction of solids are depleted (−25 to −28‰) relative to the carbonates (−11 to −20‰). We conclude that heterotrophic processes are dominant throughout the surface ecosystem, and carbon fixation may be key down channel. δ^15^N ratios ~3‰, and absence of *nifH* in extracted DNA suggest that nitrogen fixation is not occurring in sediments. However, the presence of *narG* and *nirS* at most locations and in enrichments indicates genomic potential for nitrate and nitrite reduction. This small seep with shallow run-off is likely ephemeral, but abundant preserved microterracettes in the outflow and the surrounding area suggest it has been present for some time. This site and others like it present an opportunity for investigations of preserved deep biosphere signatures, and subsurface-surface interactions.

## Introduction

### Terrestrial serpentinizing fluid seeps

The habitable subsurface represents a largely unknown and unexplored habitat. Some estimates have placed the extent of this habitat in the Earth's crust as deep as 5–10 km, with a capacity to accommodate up to 2 × 10^14^ tons of biomass (Gold, 1992; Whitman et al., 1998; Jorgensen, 2012; Kallmeyer et al., 2012). The high end of these estimates would represent more biomass and biodiversity than in Earth's surface environments. The role that subsurface habitats play in global biogeochemical cycling has yet to be described, but is certain to be substantial.

A major challenge in studying the deep subsurface is difficult access. Deep drilling on continents and on the seafloor is expensive. Terrestrial expressions of the deep biosphere, such as serpentinizing springs issuing from exposures of ophiolites, are easy and cheap to access in comparison to their marine counterparts. Serpentinization is the aqueous alteration of ultramafic rocks, which causes pervasive alteration in solids, yields distinctive aqueous geochemistry, evolves gases such as H_2_ and CH_4_, and furnishes chemical energy sufficient to drive chemosynthetic life. Recent reviews outline the process of serpentinization and the habitats created in the subsurface (e.g., Sleep et al., 2011; Schrenk et al., 2013).

It has been proposed that serpentinization may have provided critical energy, carbon, and support for life on the Early Earth (Sleep et al., 2004). These processes likely continue to support life in “extreme” subsurface settings on the modern Earth. Olivine-bearing mafic rocks similar to those on Earth exist at or below the Martian surface (Hoefen et al., 2003; McSween et al., 2004; Lipps and Reiboldt, 2005), and in fact serpentine phases on the surface of Mars have been identified definitively (Ehlmann et al., 2009). It has been argued that the very reducing mineral assemblage associated with ultramafic rocks and serpentinization presents opportunity for chemoautotrophic metabolisms on both Earth and Mars (Hoefen et al., 2003; Sleep et al., 2004; Schulte et al., 2006). Understanding the subsurface habitability of terrestrial serpentine terrains will ground-truth the search for life in ultramafic rock complexes beyond modern Earth.

This report characterizes a high pH fluid seep found in the Tekirova ophiolite region of southern Turkey, where active serpentinization brings H_2_ and CH_4_ in contact with formation fluids, energizing the subsurface biosphere. The purpose of this work is to evaluate the geochemistry of the environments present at the site, relate the characteristics of the fluids to other known terrestrial serpentinizing fluid seeps, and present data that highlight the microbiology and potential for carbon and nitrogen cycling at the site to develop an image of the nature of this unique ecosystem.

### The Tekirova ophiolite

At the southern coast of Turkey near the town of Çıralı, a large exposure of the Tekirova ophiolite hosts one of the few known terrestrial methane seeps in the world, thought to have been burning continuously for ~2000 years (Hosgörmez, 2007). This site—known as “Chimera” in previous reports (Hosgörmez, 2007; Hosgörmez et al., 2008; Etiope et al., 2011) but known locally as “Yanartaş” (meaning “flaming rock” in Turkish)—consists of several small gas seeps occurring along fault lines and secondary fractures in the rock with significant diffuse seepage of hydrocarbons noted in the vicinity of the larger, actively burning vents (Hosgörmez et al., 2008; Etiope et al., 2011). Similar large, terrestrial methane seeps have been located in Oman (Barnes et al., 1978), New Zealand (Lyon and Giggenbach, 1990), and the Philippines (Abrajano et al., 1988). The Pamphylian suture represents the boundary between the southern portion of the Anatolide-Tauride Block and accreted oceanic crustal material and parallels the location of diffuse methane seepage. At Yanartaş, serpentinized harzburgite is exposed at the surface (Bağci et al., 2006) and serpentinite of the same complex (Antalya Complex) is exposed nearby. This site is likely the best example of active serpentinization in the area (Etiope et al., 2011). Mineral composition of the Tekirova ophiolite includes brucite, hydromagnesite, serpentine, chrysolite, olivine, magnetite, lizardite, dunite, gersdorfite, aragonite, and calcite (Hosgörmez, 2007).

### Gas and fluid seeps at Yanartaş

The concentrations and isotopic compositions of C1–C6 gases, CO_2_, N_2_, and H_2_ at Yanartaş have been previously reported (Hosgörmez, 2007; Hosgörmez et al., 2008; Etiope et al., 2011), including some areas of diffuse seepage in addition to the discrete vent locations (Etiope et al., 2011). Typical measured CH_4_ and H_2_ contents were ~87 and 9.8 vol%, respectively, with the bulk of the remaining gas being N_2_ (Etiope et al., 2011). The δ^13^C ratio of CH_4_ was reported as −12.51, and it was determined that the seeps are releasing gases of mixed thermogenic and abiotic origins (the latter related to low temperature serpentinization in the Tekirova ophiolite unit) (Etiope et al., 2011). Further, the total CH_4_ seepage (both burning and non-burning locations) was estimated to equal 150–190 t year^−1^, and >27,000 L of gas per hour (Etiope et al., 2011). Previous reports have specified that no fluid seeps were present at Yanartaş.

Our visit to the Yanartaş field site in February, 2012 confirmed active gas seeps along the exposure, many of which were ignited. In addition, a small fluid seep was discovered. As previous reports have not mentioned this seep, or have stated that no fluid seeps are present, it is likely the seep is ephemeral. This report provides the first geomicrobiological dataset for this site. Fluid at the source of the seep, and several locations down a forked outflow channel were sampled. On-site analyses included temperature, conductivity, and pH measurements. Fluids were analyzed for major cation, anion, and trace element composition, as well as δ^13^C isotopic ratios of dissolved organic and inorganic carbon. Solid materials (biomass, biominerals, and sediment) were collected as well, and microbiological analyses included δ^13^C isotopic ratios of total and organic carbon, δ^15^N isotopic ratios, and scanning electron microscopy (SEM); mineralogical and chemical analysis was conducted by X-ray diffractometry (XRD) and energy dispersive X-ray spectroscopy (EDX). Sediment and fluids were used to inoculate enrichment media to investigate the potential for carbon and nitrogen cycling in the surface communities. Environmental samples and enrichments were screened for presence/absence of genes associated with nitrogen cycling.

## Methods

### Field measurements

Temperature and pH were measured at the time of sample collection using hand-held meters calibrated in the field (YSI 30 and Orion 290A plus meters). Only sites YT-0m and YT-S8.8m had enough fluid for measurement. Sediment temperature at YT-0m was measured with a probe style thermometer by pushing it into the sediment, avoiding the actively burning gas source.

### Sample collection

Fluid samples were collected at sites YT-0m and YT-S8.8m only; the depth of fluid at the other sites was only a few mm and thus was not conducive to bulk collection. Fluids were collected by slowly drawing into a 60 ml syringe, and combined to fill fully a 500 ml Nalgene bottle. Fluid was filtered through Sterivex filters (EMD Millipore, Billerica, MA, USA) into bottles specifically prepared for each fluid analysis.

Solid samples (biofilms, biominerals, and sediments) were collected using sterile scoops and placed into sterile Whirlpac bags. Samples intended for nucleic acid extraction were frozen at −20°C on return to the facility, ~2 h after collection. All other samples were kept at 4°C until analysis.

### Analysis of cations/anions in fluids

Samples for analysis of cations and anions were filtered into 60 ml Nalgene bottles prepared by soaking overnight in an acid bath. Bottles were kept frozen until analysis. Analyses by ion chromatography were as described previously, at Arizona State University (ASU) (Shock et al., 2010; Meyer-Dombard et al., 2011).

### Analysis of trace elements in fluids

Sixty mL Nalgene sample bottles were soaked in 10% HNO_3_ (trace metal grade) for 1–3 days, rinsed with deionized water, and sealed in the lab before travel. Samples were filtered through a series of 1.0, 0.8, and 0.25 μm filters and acidified to pH <2 with HNO_3_. Analysis was performed using a Finnigan MAT (Thermo Electron) Element 2 single-collector double-focusing magnetic sector inductively coupled plasma mass spectrometer (ICP-MS) at ASU. Uncertainties are one standard deviation for minor (3%) and trace (5%) elements. Accuracy and precision were determined with river water standard reference materials NIST 1640 and NRC SLRS4; measured and certified values for standards were within quoted uncertainties.

### Analysis of dissolved organic and inorganic carbon in fluids

Fluid samples were collected for analysis of dissolved organic carbon (DOC) and dissolved inorganic carbon (DIC) composition. Samples were collected in amber I-CHEM bottles with either butyl/teflon (DIC) or silicon/Teflon (DOC) septa. Bottles were filled fully with no air bubbles, and kept at room temperature until analysis. DIC bottles and septa were soaked in 10% HCl overnight. DOC bottles were combusted at 500°C overnight, and 100 μl of ASC grade 85% phosphoric acid was added prior to adding sample. DOC and DIC concentrations were measured with an OI Analytical Model 1010 Wet Oxidation Total Organic Carbon (TOC) Analyzer at ASU. Samples were reacted with either phosphoric acid (DIC) or sodium persulfate (DOC), and resulting CO_2_ was analyzed by continuous flow into a Thermo Delta^Plus^ Advantage mass spectrometer. Three glycine working standards characterized with USGS40 and USGS41 isotopic reference materials were used (low: δ^13^C = −39.64‰, δ^15^N = 1.35‰; mid: δ^13^C = −8.36‰, δ^15^N = 27.9‰; and high: δ^13^C = 15.67‰, δ^15^N = 51.8‰) that encompass expected isotopic variations.

### Analysis of oxygen and hydrogen stable isotopes in fluids

Samples were collected in 30 ml glass bottles (Qorpak, Bridgeville, PA, USA) by filling fully to the top and ensuring no air bubble was entrained. Isotopes of hydrogen and oxygen were measured by Off-Axis Integrated Cavity Output Spectroscopy (OA-ICOS) on a Los Gatos Research (LGR) DLT-100 accompanied by a CTC PAL autosampler at ASU. Ten injections of 930 nL of each sample were analyzed, and the first five runs of each were disregarded to adjust for sample carry over memory effect between sample analyses. Instrument drift and normalization to VSMOW were addressed as found in van Geldern and Barth (2012).

### Analysis of solids by XRD

Mineral composition was determined by x-ray diffractometry (XRD) on a Terra portable x-ray diffractometer, distributed by Olympus (Auburndale, MA, formerly InXitu). Standard operating procedures engage a Co x-ray source and a cooled charge-coupled device (CCD) detector arranged in transmission geometry with the sample, with angular range of 5°–50° 2θ with <0.35° 2θ resolution (cf. Blake et al., 2012). X-ray tube voltage is typically 30 kV, with a power of 10 W, a step size of 0.05°, and an exposure time of 10 s per step. Total run time comprises 1000 exposures, requiring about 75 min total run time. Prior to analyses, samples are powdered using a percussion mortar or agate mortar and pestle; when necessary a Dremel manual drill was used to subsample grains of interest. Powders are passed through a standard 150 μm sieve (or 100-mesh). About 15 mg of powdered material is transferred with a spatula to the inlet hopper of the standard sample vibration chamber, which continuously mixes the powdered sample for the duration of the analysis. Interpretation of diffractograms is conducted with XPowder software, which is a commercially available peak search-and-match program that queries the PDF2 database for reference mineral peak information. Typically XRD accounts for mineral phases present at levels ≥~5% of the sample volume.

### Analysis of carbon and nitrogen isotopic ratios in solids

Biofilm and sediment samples were freeze dried and then ground with an agate mortar and pestle until powdered uniformly. They were weighed, placed in tin capsules, sealed, and analyzed using a Costech Model ECS 4010 Elemental Analyzer (Costech Analytical Technologies Inc., Valencia, CA, USA) coupled to a Thermo Delta^plus^ Advantage Isotope Ratio Mass Spectrometer (EA irMS) (Thermo Fisher Scientific Inc., Waltham, MA, USA) at ASU. Three glycine working standards that spanned the expected isotopic variations were used to standardize data, as described in Section Analysis of Dissolved Organic and Inorganic Carbon in Fluids. Linearity checks were performed using NIST 2710 (Montana Soil).

### Analysis and imaging of solids by SEM/EDX

Biofilm and sediment samples collected for SEM/EDX analysis were freeze dried and mounted onto stainless steel stubs using carbon tape (Electron Microscopy Sciences, Hatfield, PA, USA). Sample stubs were analyzed using a Hitachi S-3000N Variable Pressure SEM (Hitachi High-Technologies Corporation, Tokyo, Japan) equipped with an Oxford INCA EDS with light element X-ray detector at the University of Illinois at Chicago (UIC). Samples were imaged uncoated using a backscattered electron detector in variable pressure mode. The instrument has a backscattered electron image resolution of 5.0 nm at 25 kV, a magnification range of 15X to ~300,000X, variable pressure range from 1 to 270 Pa, and accelerating voltages of 0.3–30 kV.

### Enrichment culturing

Geochemical data (Table 1) were used to design growth media that mimicked site-specific chemistry. Growth media for each site consisted of 1 L base solution (0.01 g L^−1^ KCl, 0.6 g L^−1^ MgCl_2_^*^6H_2_O, 0.2 g L^−1^ NaHCO_3_, 3 mg L^−1^ NH_4_Cl, 3 mg L^−1^ NaNO_3_, 0.3 mg L^−1^ K_2_HPO_4_), amended with 10 ml of trace element solution (0.25 mg L^−1^ CoCl_2_^*^6H_2_O; 1.3 mg L^−1^ MnCl_2_^*^4H_2_O; 0.5 mg L^−1^ CuSO_4_^*^5H_2_O; 0.5 mg L^−1^ Na_2_ MoO_4_^*^2H_2_O; 0.5 mg L^−1^ NiCl_2_^*^6H_2_O; 4.1 mg L^−1^ ZnSO_4_^*^7H_2_O; 4.4 mg L^−1^ SrCl_2_^*^6H_2_O; 0.6 mg L^−1^ VOSO_4_^*^3.5H_2_O; 0.005 mg L^−1^ CdSO_4_^*^8/3H_2_O; 0.1 mg L^−1^ RbCl; 0.3 mg L^−1^ BaCl_2_^*^2H_2_O), 5 ml of CaCl_2_ solution (0.4 mg 200 ml^−1^ CaCl_2_^*^2H_2_O), and 2 ml of Fe(II)-EDTA solution (3.6 mg 200 ml^−1^ of FeSO_4_^*^7H_2_O and Na^−^_2_ EDTA). All amendments were either autoclaved separately or applied through a 0.2 μm Whatman filter. The pH was adjusted to the desired value just prior to autoclaving. pH values of 8.5, 9.5, and 10.5 were tested. A variety of organic buffers (HEPES, CAPS) and inorganic buffers (e.g., HCO^−^_3_/CO^2−^_3_) were employed to maintain the solution pH throughout each cultivation experiment. The type of buffer used was dependent on the target pH of the media and whether organic buffers would influence sub-culturing of target populations (e.g., organic buffers were omitted when preparing growth media for strict chemoautotrophs). Organic buffers were added at 3 g L^−1^, while the carbonate buffer system included 2 g L^−1^ Na_2_CO_3_ and 0.5 g L^−1^ NaHCO_3_. Several iterations of growth media were prepared targeting different metabolic pathways. These were; yeast extract-peptone (3 g L^−1^each), organic acids (0.14 g L^−1^ each formate, acetate, propionate), sugars (5 g L^−1^ each sucrose, lactose, glucose), nitrate reduction (0.03 g L^−1^ NaNO_3_), sulfate reduction (0.2 g L^−1^ Na_2_SO_4_, excluded any nitrate salts), and ferric iron reduction (0.3 g L^−1^ FeCl_3_, without any sulfate or nitrate salts). These media were inoculated with a slurry made from spring fluids and sediments taken from site YT-N3.6m, and incubated at 30°C, 40°C, and 50°C to obtain enrichments. Cultures were placed under either an 80:20 N_2_:H_2_ headspace for anaerobic cultures, or filtered air for aerobic cultures. Cell growth was verified via phase contrast and epifluorescent microscopy as previously described (Meyer-Dombard et al., 2012).

**Table 1:** **Field data and geochemistry of fluids collected at YT-0m and YT-S8.8m along the outflow of the Yanartaş fluid seep**.

	**YT-0m**	**YT-S8.8m**
**ON-SITE DATA**		
Fluid temperature (°C)	18.5	19.1
Sediment temperature (°C)	65	n.d.
pH	11.95	9.4
Conductivity μS	1357	1359
**MAJOR IONS**		
Cl^−^ (ppm)	18.3	24.31
Br^−^ (ppm)	0.05	0.04
SO^−2^_4_ (ppm)	8.0	29.1
PO^−3^_4_ (ppm)	0.0	0.0
NO^−^_2_ (ppm)	0.0	0.0
NO^−^_3_ (ppm)	0.05	0.09
Na^+^ (ppm)	11.48	12.25
NH^+^_4_ (ppm)	0.57	0.0
K^+^ (ppm)	2.82	6.90
Mg^+2^ (ppm)	0.69	68.56
Ca^+2^ (ppm)	138.82	10.98
Ca:Mg	201.2	0.16
**DISSOLVED CARBON**		
DOC (ppmC)	n.d.	5.2
δ^13^C DOC (‰ VPDB)	n.d.	−22.75 ± 0.43
DIC (ppmC)	n.d.	50.7
δ^13^C DIC (‰ VPDB)	n.d.	−11.8 ± 0.49
**ISOTOPES**		
δ^18^O	−4.29 ± 0.06	−2.89 ± 0.1
δD	−23.05 ± 0.46	−17.71 ± 0.92
**TRACE ELEMENTS**		
Li (ppb)	23.6	24.3
Al (ppb)	10.8	21.8
P (ppb)	5.9	77
V (ppb)	0.24	1.45
Cr (ppb)	0.08	1.68
Mn (ppb)	0.06	3.6
Fe (ppb)	1.5	59
Co (ppb)	0.02	0.56
Ni (ppb)	0.79	14.7
Cu (ppb)	0.36	1.16
Zn (ppb)	2.93	9.4
Ga (ppb)	0.18	0.03
As (ppb)	0.47	0.61
Rb (ppb)	0.56	0.97
Sr (ppb)	253	14.4
Mo (ppb)	0.13	0.46
Cd (ppb)	0.03	0.02
Cs (ppb)	0.13	0.19
Ba (ppb)	5.25	1.6

n.d., not determined.

### DNA extraction from environmental samples and enrichment cultures

Actively growing enrichments were spun down in a centrifuge and pellets were collected using a sterile spatula. DNA was extracted from the cell pellets using the Fast DNA Spin Kit for Soil (MP Biomedicals, Santa Ana, CA, USA) following the manufacturer's instructions (but substituting the cell pellet for a soil sample).

Environmental DNA was extracted from biofilm and sediment samples using the MoBio PowerBiofilm DNA Isolation Kit (MO BIO Laboratories, Inc., Carlsbad, CA, USA). Following manufacturer's protocol, approximately 200 mg of sediment were weighed into the PowerBiofilm Bead Tubes; final extracts were stored in an −80°C freezer until further analysis.

### Screening of 16S rRNA and ecosystem function genes

16S rRNA and ecosystem function genes related to the nitrogen cycle were amplified from extracted DNA for select enrichment cultures, based on the quality of the DNA extracted and ability to clone/sequence the genes. In addition, DNA extracted from environmental samples was screened for the presence of the same nitrogen cycle genes. These latter environmental surveys were presence/absence only and sequencing of individual genes was not attempted.

DNA extracts were amplified by the polymerase chain reaction (PCR) with 27F and 1492R bacterial 16S rRNA primers, using recipes and protocols as previously described (Meyer-Dombard et al., 2005). For function-based gene analysis, DNA extracts were screened for the presence of nitrogenase (*nifH*), respiratory nitrate reductase (*narG*), nitrite reductase, (*nirS/nirK*), and nitric oxide reductase (*norB*). The following primers were used; primer sequences can be found in the indicated literature. *NifH* primers: nifH1F and nifH1R (Mehta et al., 2003) at 100 pmol μL^−1^, *nirK* primers: FlaCu and R3Cu (Throback et al., 2004) at 50 pmol μL^−1^, *nirS* primers: Cd3AF and R3cd (Throback et al., 2004) at 50 pmol μL^−1^, and *norB* primers: cnorB2F and cnorB6R (Braker and Tiedje, 2003) at 50 pmol μL^−1^. For all primer sets, 2 μL of template DNA were added to 18 μL of PCR mix containing 10 μL DreamTaq™ Green PCR Master Mix (2×) (Fermentas Inc., Glen Burnie, MD, USA), 6.5 μL of nuclease-free water, 0.5 μL of 50× bovine serum albumin (BSA), 0.5 μL of each primer. PCR amplification was conducted on a S1000 model thermal cycler (BioRad, Hercules, CA, USA).

Successfully amplified 16S rRNA and targeted ecosystem-function genes from select cultures were cloned using the TA TOPO Cloning Kit (Qiagen) according to the manufacturer's instructions. Clones were screened with PCR to verify fragments were of the correct size prior to sequencing at the DNA Sequencing Facility at the University of Illinois at Chicago. Similarity to known genes was determined using the Basic Local Alignment Search Tool (BLAST; Altschul et al., 1990) via the National Center for Biotechnology Information (NCBI) website. Sequences have been deposited with the National Center for Biotechnology Information (NCBI) as accession numbers KP214434-KP214442.

## Results

### Site description

**Figure 1:**
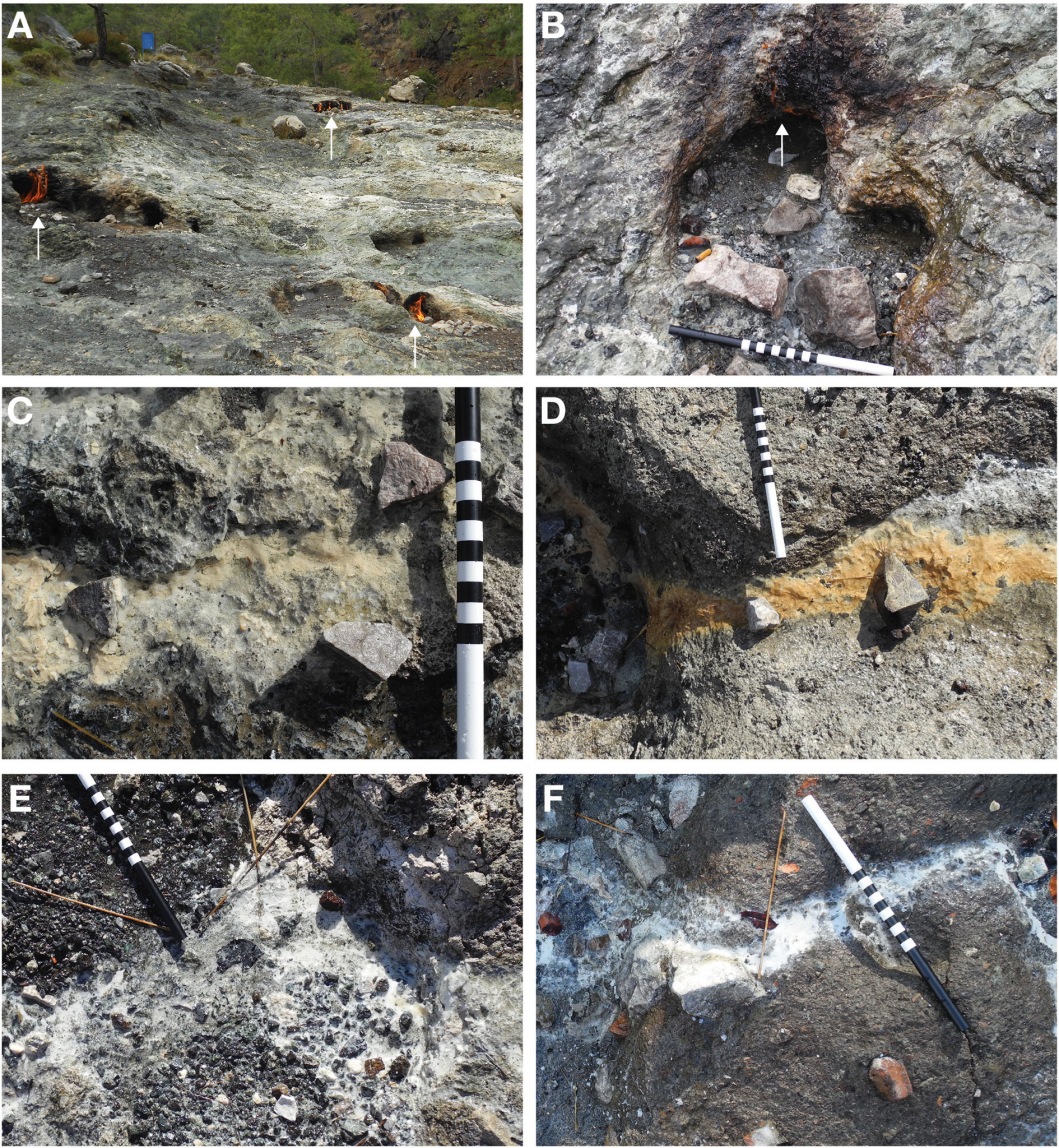
**Images of Yanartaş area gas seeps and fluid seep biofilms**. In **(B–F)**, scale is 30 cm total; 10 cm each for black, striped, and white segments. **(A)** Exposed hillside showing three (ignited) gas vents (white arrows). Blue can in upper left is a 55 gal. drum for scale. **(B)** Site YT-0m, source of fluid seep. White arrow shows small flame from the associated gas seep. Fine, black sediment was sampled from under the gas seep. **(C)** Site YT-N3.6m tan biofilm. **(D)** Site YT-N6.7m rust-colored biofilm. Break in slope in hillside can be seen toward left of frame. Preserved microteracettes are located on the vertical face (not visible in this photograph). **(E)** Site YT-S4m white biofilm. **(F)** Site YT-S8.8m white biofilm.

**Figure 2:**
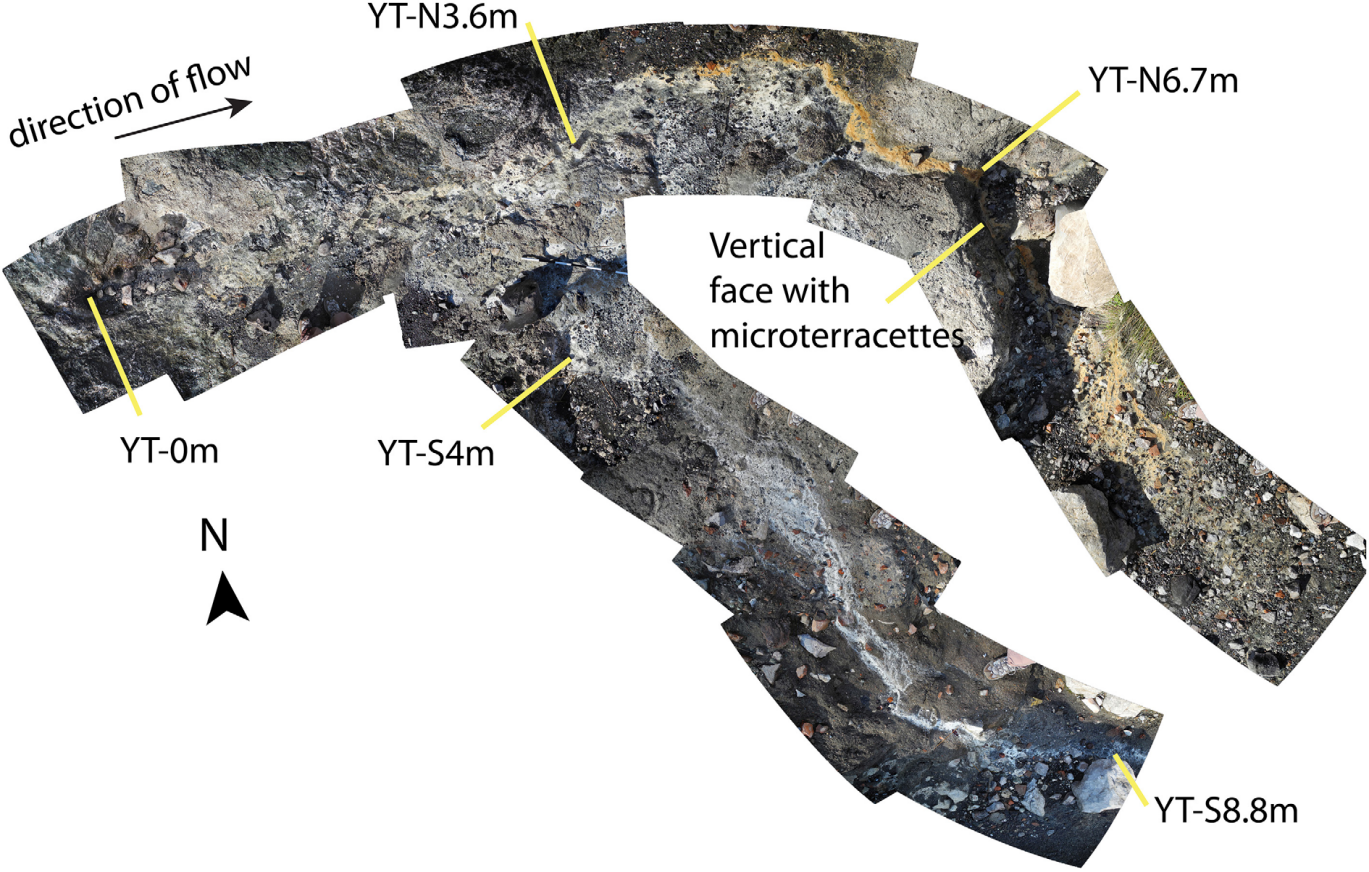
**Photomosaic collage of the sampled area of the Yanartaş fluid seep, which serves as a site map**. Source of both gas and fluid is site YT-0m. Sites on the northernmost and southernmost forks are noted by “YT-N” and “YT-S” respectively, followed by the distance downstream from the source in meters. The location of microterracettes in the northern fork notes the location where the break in the exposure's slope occurs. A scale can be seen placed at the fork of the run-off—about 70 cm are visible, where each white and black section are 10 cm long.

The seeps at Yanartaş are accessible via a groomed path used by the tourism venue on site. The area is an exposed, primarily unvegetated hillside facing roughly East, surrounded by evergreen forest (Figure 1A). Numerous gas vents are scattered around the exposure, tending to follow fractures and perhaps faults. The fluid seep emanates from a small gas vent location, ~15 cm in diameter, actively burning (Figure 1B). The sediment under the active flame is fine and black. Seep fluid runs down the slope, splitting into a Y-shaped outflow channel about 3 m downstream (Figure 2). Around 3 m, mineral precipitates are abundant. The northernmost fork contained precipitates that were of rust-orange color, beginning ~0.5 m from the outflow fork (Figures 1C,D, 2). The outflow immediately prior to the split, and continuing down the southernmost fork was coated in tan or white precipitates. The texture of the precipitates varied from solid and crusty to soft and gelatinous. These latter are presumed to incorporate biofilm material (e.g., Figures 1E,F). Around 7 m downstream, there is a break in slope in the hillside, creating a small vertical drop over which the fluid flows. Here, there are preserved microterracette structures, suggesting that the fluid has progressed in this general direction for long enough to generate mineral deposits (Figure 1D). Similar microterracettes are located elsewhere around the gas seep exposures, further suggesting that additional fluid seeps have been present in the past, and may continue in a similar ephemeral manner to the one described here.

### Fluid geochemistry

Analysis of the geochemistry of the fluids and solids collected from Yanartaş allows comparison to other similar sites worldwide and serves as a backdrop upon which to consider the nature of these ecosystems. Due to the small volume of fluid emanating from the seep's source, and shallow depth of the outflow (a few mm), fluid samples were only obtained from two locations at Yanartaş—at the source (YT-0m) and the end of the southernmost outflow channel (YT-S8.8m). Full geochemical results are reported in Table 1. The temperature and conductivity of fluids are largely invariable downstream. Temperature in the sediments under the ignited gas source, as measured by a probe-style thermometer pushed ~10 cm into the sediments, is 65°C. The pH changes dramatically downstream, measured at nearly 12 at the source to ~9.5 at the end of the outflow channel.

Major ions of highest concentration include Cl^−^, SO^−2^_4_, Na^+^, Mg^+2^, and Ca^+2^. Trace elements of highest concentration for both samples are Li, Al, P, and Sr, while site YT-S8.8m also has elevated Fe, Ni, and Zn concentrations. In general, site YT-S8.8m shows higher concentrations of trace elements than the source of the seep at 0 m.

Concentrations of dissolved organic and inorganic carbon (DOC and DIC, respectively) are also given in Table 1 for site YT-S8.8m—fluid volume at site YT-0m was too low to obtain sufficient sample for these analyses.

### Geobiology and mineralogy of solids

**Figure 3:**
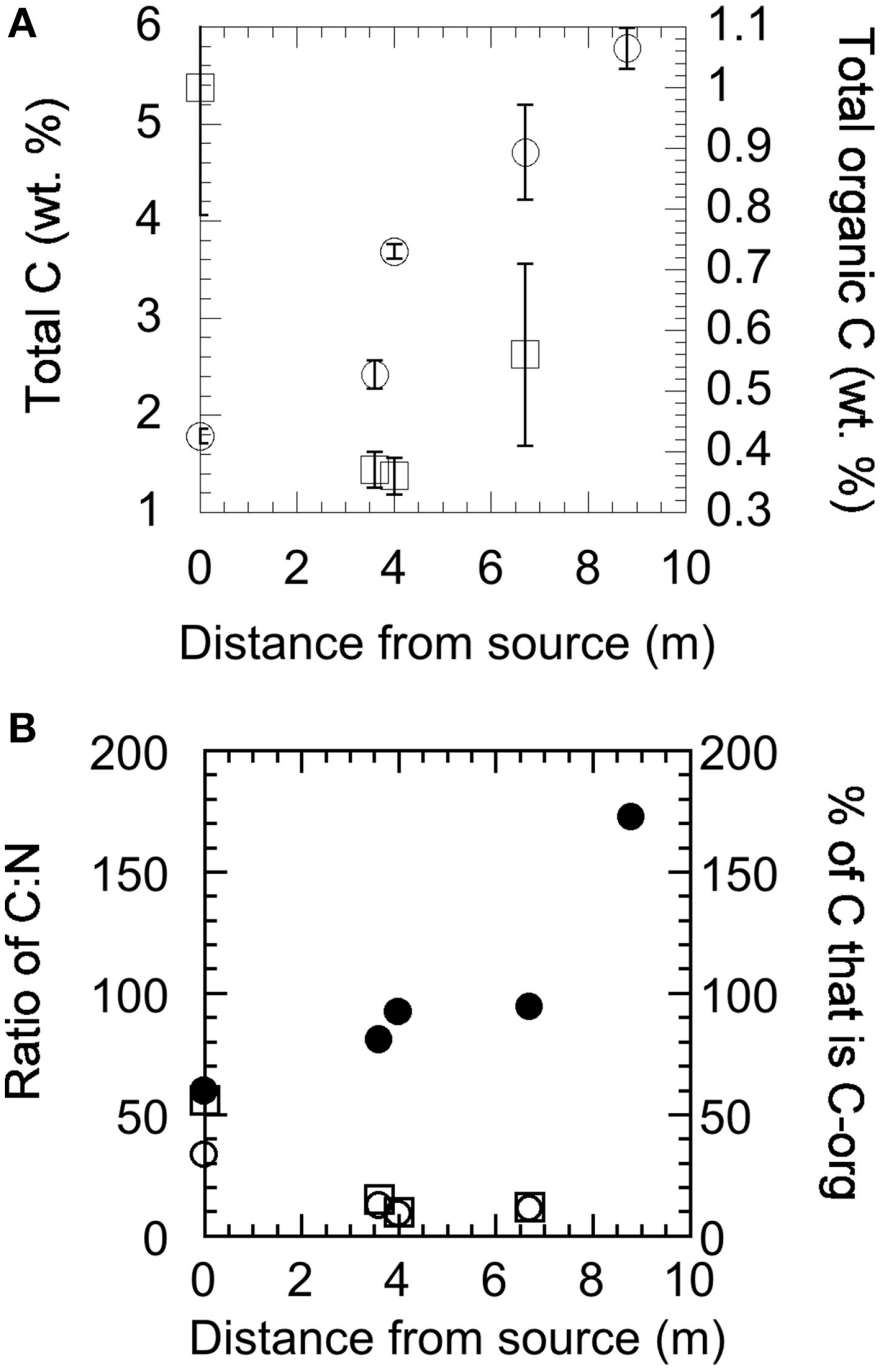
**Total carbon, total organic carbon, and total nitrogen content of solid materials collected at the source (YT-0m) and down the two outflow channels as analyzed by EA-irMS. (A)** Total carbon (open circles) and total organic carbon (open squares) as a function of downstream sampling. **(B)** The ratio of total carbon to total nitrogen (solid circles) and organic carbon to total nitrogen (open circles), and the percentage of carbon in each sample that is organic carbon (open squares).

Solids collected at each site were analyzed for mineralogical and isotopic composition by XRD, EDX, and EA-irMS. Carbon and nitrogen composition, given as wt% of sample analyzed, are shown in Figure 3. The total carbon content increases as a function of distance from the source of the fluid (Figure 3A) and while the total amount of organic carbon is highest at the source, the proportion of total carbon to organic carbon increases downstream (Figure 3B). There was too little organic carbon at site YT-S8.8m to be analyzed. The nitrogen content of the samples tracks the organic carbon content closely (Figure 3B). The isotopic composition of total carbon in the solid materials collected from Yanartaş is −20.11 ± 0.53‰ at the seep source, becoming more enriched (−11 to −12‰) at downstream locations (Table 2). This signature is consistent with the isotopic composition in DIC found in the fluids at YT-S8.8m, which is −11.8‰ (Table 1). The depleted signal at the source is likely due to the influence of organic carbon in the sample, which is −27.7 ± 0.53‰ at the source. Organic carbon becomes slightly more enriched downstream, ~-25‰ in both outflow channels. This is slightly depleted relative to the isotopic composition of DOC, which is −22.75‰ (Table 1). The isotopic composition of nitrogen in the solid samples is enriched relative to that in air, at ~3‰ at all locations within analytical error.

**Table 2:** **Geochemistry of solids collected along the outflow of the Yanartaş fluid seep**.

	**YT-0m**	**YT-N3.6m**	**YT-N6.7m**	**YT-S4m**	**YT-S8.8m**
**C AND N BY EA-irMS**					
Total C (wt.%)	1.79	2.42	4.71	3.69	5.78
δ^13^C of total C (‰ VPDB)	−20.11 ± 0.53	−11.45 ± 0.3	−11.05 ± 0.2	−11.44 ± 0.2	−12.09 ± 0.08
Total C-org (wt.%)	1	0.37	0.56	0.36	n.m.
δ^13^C C-org (‰ VPDB)	−27.7 ± 0.77	−25.08 ± 0.2	−24.55 ± 0.3	−25.57 ± 0.3	n.m.
Total N (wt.%)	0.03	0.03	0.05	0.04	0.03
δ^15^N total N (‰ vs. air)	2.27 ± 0.86	3.38 ± 0.22	3.23 ± 0.84	2.99 ± 0.43	2.99 ± 0.05
**ELEMENTAL COMPOSITION BY EDX (wt.%)**					
C	2.7	11.6	10.6	6.6	16.5
O	35.0	35.9	35.0	38.2	36.8
Mg	8.5	3.2	0.5	15.6	5.9
Si	24.3	2.7	n.d.	15.1	1.4
Cl	1.1	1.7	0.6	0.9	1.4
Ca	4.5	44.9	54.0	17.9	26.1
Fe	19.4	n.d.	n.d.	4.9	n.d.
**MINERALOGY BY XRD**					
Serpentine	✔		✔		
Chlorite	✔				
Montmorillonite	✔	✔	✔	✔	✔
Calcite	✔		✔		
Aragonite	✔		✔		

n.m., not measured; n.d., not detected.

Results of mineralogical analysis can be found in Table 2. The mineral assemblage at the source of the fluid seep contains serpentine and chlorite (products of the serpentinization process) as well as calcite, aragonite, and montmorillonite. Most downstream locations are dominated by montmorillonite, with the exception of site YT-N6.7m, which also contains serpentine, calcite, and aragonite.

### SEM

**Figure 4:**
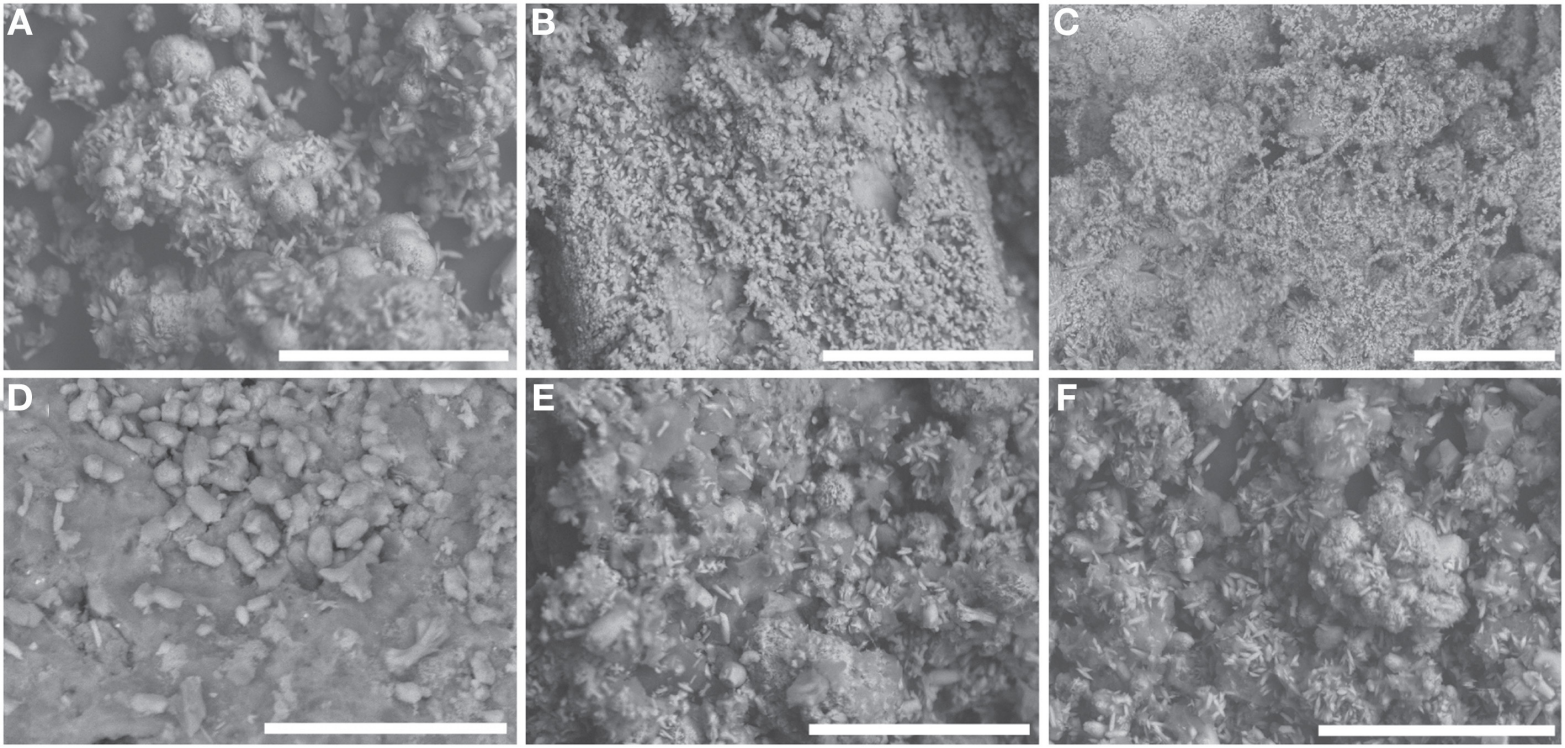
**SEM images of solid materials (minerals and biofilms) present at each sampling location**. All white scale bars are 100 μm. **(A)** YT-N3.6m, **(B)** YT-N6.7m, **(C)** YT-N6.7m showing mineralization covering filamentous microorganisms, **(D)** YT-0m, **(E)** YT-S4m, **(F)** YT-S8.8m.

At all sites, sediments and minerals are embedded with organic/biofilm material (dark gray, amorphous material in Figure 4). Sediments at YT-0m are morphologically consistent with calcite and aragonite (“fuzzy dumbbells”) (Figure 4D). The morphology of precipitates at sites YT-N3.6m, YT-S4m, and YT-S8.8m are similar, showing botryoidal forms intermingled with small needles of aragonite. Precipitates are the same scale at all three sites and can be seen sometimes coated in biofilm in Figures 4A,E,F. Site YT-N6.7m differs in both its mineralogical and morphological composition, compared to other downstream locations. Here, sediments consist of densely packed, fine needles and occasional fuzzy dumbbells (Figure 4B). Microbial filaments extending hundreds of microns (Figure 4C) can be seen at site YT-N6.7m, and these are encrusted with the same fine needles. The homogeneity of needles on the filaments and surrounding the filaments suggests abiotic precipitation of the needles rather than precipitation by the microorganisms.

### Enrichment cultures

Enrichments from Yanartaş site YT-N3.6m were dominated by fast-growing, motile, aerobic, filamentous and rod-shaped morphologies (Table 3). Growth was very poor or nonexistent in anaerobic media formulations. Growth was observed in all media with organic C sources (yeast/peptone, organic acids, sugars) up to pH 10.4, although growth was visibly stunted with increased pH (i.e., cells appeared shrunken and less mobile than at lower pH). Enrichments favored media that were pH 8–9.5. Several enrichment cultures yielded growth at moderately thermophilic temperatures (40–50°C). Typical cells were 10–12 μm long × 1 μm wide (see Supplemental Figure 1).

**Table 3:** **Results and observations of growth experiments for Yanartaş enrichments**.

**Metabolic option**	**Growth conditions**				**Observations**
	**Temp. (°C)**			**pH**	
	**30**	**40**	**50**		
Yeast and peptone (YP)	+++	+++	+++	8.5	Fastest growth at pH 8.3; reached cell densities of ~1.7 × 10^7^ cells/ml within 4 h (at pH 8.3–9.5)
	++	++	++	9.5	
	+	+	+	10.5	
Organic acids (OA)	++	++	++	8.5	Lower cell density reached than with YP as a C source; cells appear shriveled in pH 10.4 media
	+	+	+	9.5	
	+	+	+	10.5	
Sugars (S)	+	+	N/A	8.5	Lower cell density reached than with YP or OA as a C source;
	+	+	N/A	9.5	
	+	+	N/A	10.5	
NO^−^_3_ reduction (NR)	++	++	++	8.5	Growth aided by addition of C_org_
	+	+	+	9.5	
	−	−	−	10.5	
SO^2−^_4_ reduction (SR)	+	+	+	8.5	Growth aided by addition of C_org_
	+	+	+	9.5	
	−	−	−	10.5	
Fe(III) red. (IR)	+	+	+	8.5	Growth aided by addition of C_org_
	+	+	+	9.5	
	−	−	−	10.5	

All media formulations shown here were aerobic (anaerobic enrichments largely failed). The number of “+” signs indicates degree of growth success while “−” indicate no observed growth. A “+” indicates 10^3^–10^4^ cells ml^−1^; “++” indicates 10^5^–10^6^ cells ml^−1^; “+++”indicates 10^7^–10^8^ cell ml^−1^.

Several media targeting metabolic processes hypothesized to be important in fluid seeps related to deep subsurface environments were used in culturing efforts. These included autotrophic sulfate and iron reducing metabolisms. In addition, a medium targeting nitrate reduction—as a process important in both subsurface and surface ecosystems—was utilized. In comparison to media formulations focusing on heterotrophic metabolisms, media targeting autotrophic growth had only moderate success (and growth was enhanced by addition of organic carbon). Of the autotrophic formulations, the media targeting nitrate reduction was the most successful.

Enrichment cultures grown in heterotrophic formulations grew rapidly and formed extensive biofilms. A time series image of growth in the Yeast-peptone medium (pH 9.5, 50°C) taken over 4 h demonstrates the rapid development from single cells into a biofilm-supported community in this medium (Supplemental Figure 1). At *t* = 0 h, the motile rods were ~10 μm in length. Cell numbers and size increase within 1–2 h. By *t* = 3 h, cells begin to form filamentous chains, linking together end to end. At *t* = 4 h, the filaments become wider as they line up adjacent to one another, forming an interconnected web. This rapid growth pattern was observed in all cultures given organic carbon, (including organic acids and sugars, though growth was slower with these carbon sources), even after several weeks of dormancy induced by refrigeration at 4°C.

Two Yanartaş enrichments were selected for cloning and sequencing of the 16S rRNA gene, based on the quality of extracted DNA and ability to clone the PCR products. The first, grown at 50°C at pH 8.3 in media that targeted the reduction of Fe(III), contained bacteria that were most closely related to *Bacillus licheniformis* and uncultured *Thermobacillus* sp., varying in 16S rRNA gene sequence similarity in individual clones from 93 to 99% identical. Close relatives (99% 16S rRNA gene similarity over area analyzed) of *Brevibacillus limnophilus* and *Anoxybacillus flavithermus* were identified in an enrichment targeting nitrate reduction, growing at pH 9.5 at 50°C.

### Results of ecosystem-function gene surveys

Nitrogenase genes were not detected in any Yanartaş enrichments. However, we were able to amplify nitrate reductase (*narG*), nitrite reductase (*nirS*), and nitric oxide reductase (*norB*) genes using gene-specific primers from enrichments grown in pH 9.5 and pH 8.3 media. Cell growth was limited and despite multiple extraction attempts, no amplifiable DNA was obtained from enrichments grown in carbonate-buffered pH 10.4 media. PCR products successfully amplified from DNA extracted from the above mentioned enrichment grown in the nitrate reduction medium (no organic carbon added, pH 9.5, 50°C) and amplified using the *nirS* and *narG* primer sets were cloned and sequenced. Comparison to *nirS* and *narG* genes in the NCBI dataset revealed low sequence similarity to known genes. The *nirS* gene sequenced is 84% similar to a *nirS* gene isolated from a *Halomonas* sp. C8 (accession number GQ384048). The *narG* gene sequenced from DNA from the same enrichment culture is only 91% similar to that of an uncultured bacterium (accession number AY453356).

DNA extracted from biofilm/mineral deposits at each site was also subjected to the above ecosystem-functional PCR screens. These surveys were presence/absence only and sequencing of individual genes was not attempted. In the natural samples, the *nifH* gene was only detected by PCR in samples YT-N3.6m and YT-N6.7m. The *narG* and *nirS* genes were amplifiable at all sample locations.

## Discussion

### The Yanartaş fluid seep

Our data allow the interpretation of ecological and geochemical processes occurring at the Yanartaş fluid seep, and evaluation of the similarity/dissimilarity to various other terrestrial serpentinizing seeps worldwide.

**Figure 5:**
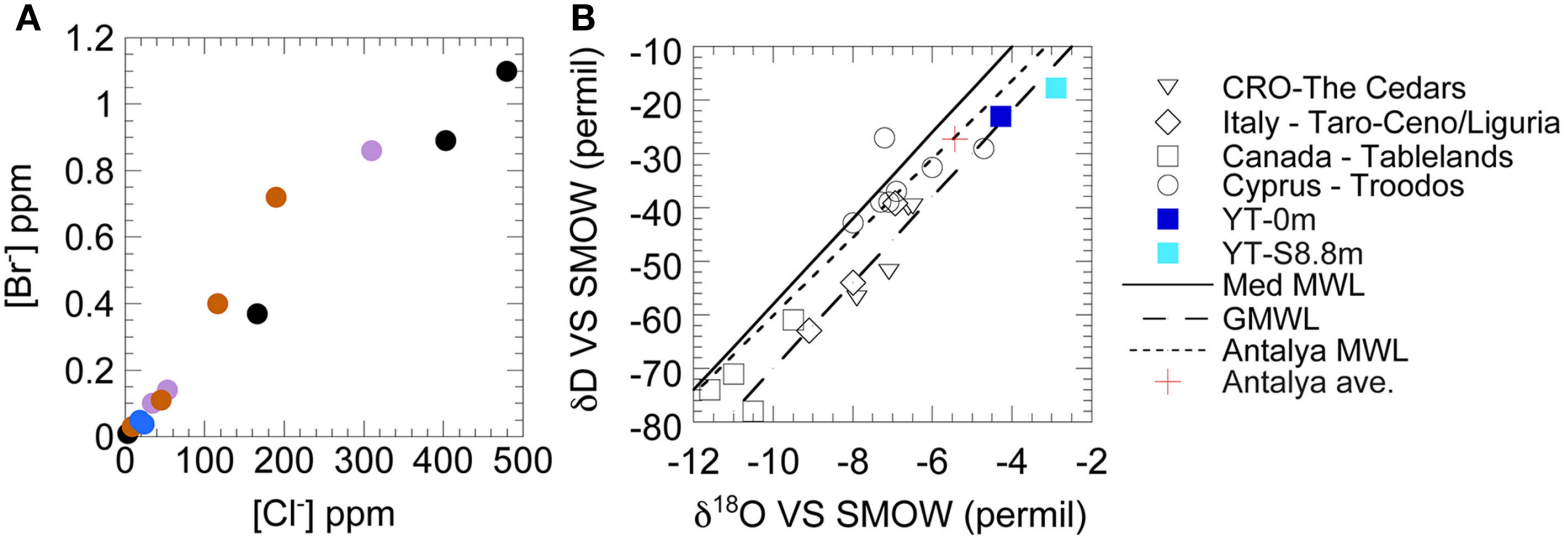
**Fluid analysis of the Yanartaş seep, shown with other globally significant serpentinizing fluids for reference**. Non-Yanartaş data are from the following reports: Barnes et al. (1978), Neal and Shand (2002), Cipolli et al. (2004), Boschetti and Toscani (2008), Okland et al. (2012), Morrill et al. (2013), Szponar et al. (2013), Tiago and Verissimo (2013). **(A)** Comparison of bromide and chloride concentrations. Purple circles, The Cedars, CA, USA; Black circles, The Tablelands, Newfoundland, Canada; Orange circles, Troodos, Cyprus, Greece; Blue circles, Yanartaş, Turkey (this work). **(B)** δ^18^O and δD of waters. Global MWL is from Craig (1961). Mediterranean MWL is from Gat and Gonfiantini (1981). The local Antalya, Turkey MWL and average values of precipitation are from Dirican et al. (2005).

Based on the low concentrations of ions and conserved elements (e.g., Cl^−^, Figure 5A), together with the isotopic composition (Figure 5B) of the source fluid, the Yanartaş fluid seep is of meteoric origins, despite its coastal location. Yanartaş fluids are slightly impacted by reaction with bedrock, sitting just to the right of the local meteoric water line (MWL) and averaged local precipitation (based on data from Antalya, Turkey—Dirican et al., 2005) (Figure 5B). The presence of relict flow paths and preserved microterracettes suggest that the fluid seep is ephemeral, but has been present for a long period of time. The possible intermittent nature of the flow is consistent with observations in previous reports that stated that there was a lack of springs in the area (Etiope et al., 2011).

**Figure 6:**
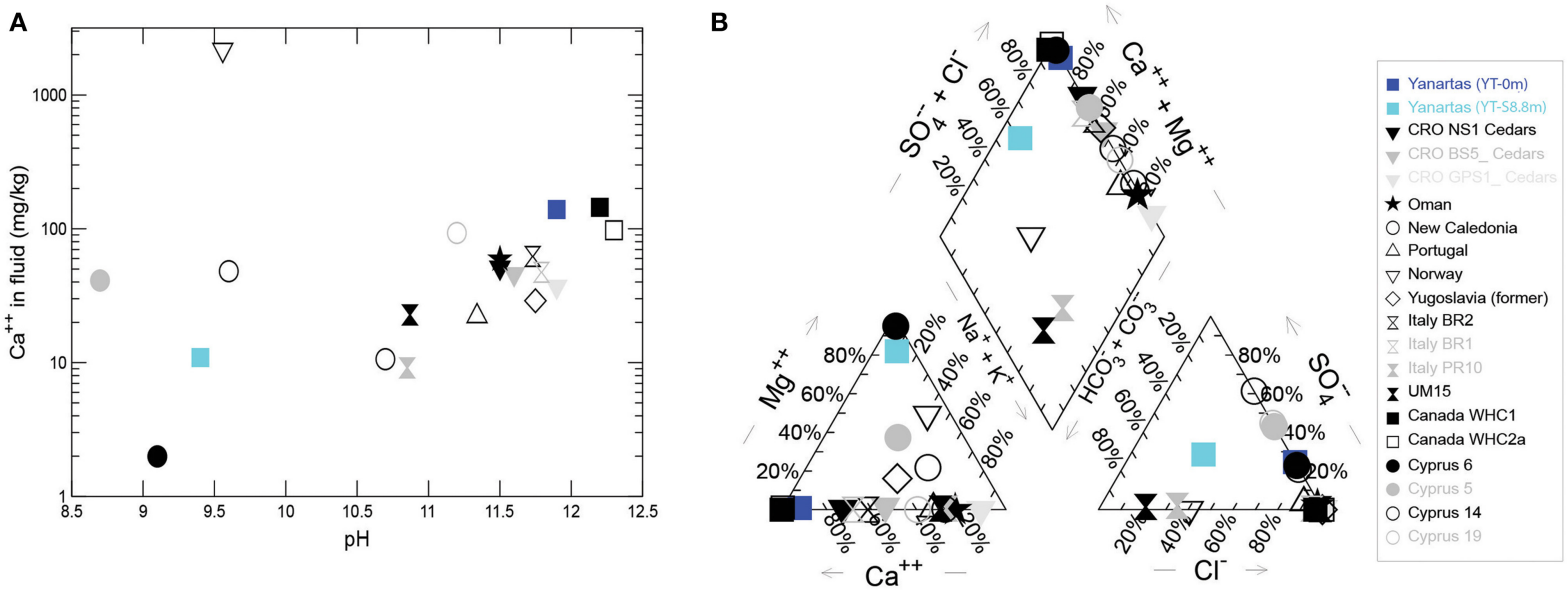
**(A)** Range of [Ca^+2^] and pH values found in serpentinizing seeps worldwide. **(B)** Piper plot showing relationships in fluid composition among the same seeps. Non-Yanartaş data are from the following reports: Barnes et al. (1978), Neal and Shand (2002), Cipolli et al. (2004), Boschetti and Toscani (2008), Okland et al. (2012), Morrill et al. (2013), Szponar et al. (2013), Tiago and Verissimo (2013).

YT-0m fluids are extremely high pH and [Ca^+2^], with negligible DIC (Figures 5A,C, 6, Table 1); this water is a classic Ca^+2^-OH^−^ type solution that is generally associated with active serpentinization (Barnes et al., 1967; Neal and Shand, 2002). Elevated Fe, Cr, Mn, Co, and Ni at YT-S8.8m co-occur with elevated Mg, suggesting that water at this site is dominated by a Mg^+2^-HCO^−^_3_ type water produced after the source fluid has passed over and weathered serpentinite/serpentine soils, accumulating products of chemical weathering (Table 1, Figure 6).

Figures 5, 6 allow comparison of Yanartaş fluids with other serpentinizing seeps worldwide. Fluid emanating from the source at Yanartaş, (YT-0m), most closely resembles that found in seeps at the Tablelands Winterhouse Creek location sampling the “ultrabasic endmember” of the seep system (Szponar et al., 2013). Figure 6 shows the wide range of pH and [Ca^+2^] featured by serpentinizing seeps; site YT-S8.8m falls within this range at the lower end of both variables, most similar to several sites in Cyprus (Neal and Shand, 2002). Site YT-0m falls within the cluster of Ca^+2^-OH^−^ type waters in Figure 6 and records some of the highest [Ca^+2^], and lowest [Mg^+2^] values in the terrestrial serpentinizing seep literature (Figure 6). Future cataloging of the microbial communities from the five Yanartaş sample locations will allow further comparison with the Tablelands and Cyprus ecosystems.

Fluid emanating from the source at pH 11.9 decreases in pH as it flows down the outflow channels. Given the very low water volume at the source and down the outflow, and lack of net change in conductivity along the flow path, it is not likely that the pH decrease is caused by dilution with meteoric water. The prevalence of montmorillonite across the sampled solids suggests that fine suspensions of this and similar clay minerals may be causing the dramatic pH change: edge charge development on montmorillonite particles may cause OH^−^ to associate with surfaces, decreasing the activity of OH^−^ (c.f., Tombacz and Szekeres, 2004). Neither brucite nor portlandite are indicated in XRD data, but precipitation of fine hydroxide minerals would also draw down the concentration of OH^−^.

The mineralogy at the source (Table 2, Figure 4) suggests that precipitation of calcite and aragonite occur immediately in the small pool that collects near the gas vent, and this process continues as the fluid moves down channel and more atmospheric carbon is incorporated. The [Ca^+2^] at the end of the outflow is low, relative to that at the source (Table 1), due to its removal by precipitation of calcite and aragonite along the way (Table 2, Figure 4). The primary mineral found in all outflow channel locations except for YT-N6.7m is montmorillonite, suggesting that weathering is playing a larger role than precipitation down channel. This is also consistent with the ephemeral nature of the system. It is likely that at times of higher flow, the precipitation of calcite/aragonite increases—this is further suggested by the presence of microterracettes on the slope where fluid was not flowing at the time of sampling. These observations are significant for two reasons; first, they provide evidence that the nature of the fluids, reaction surfaces (such as clays and calcite), and availability of resources change down channel, and second, they provide evidence that entombment of micro and molecular fossils is more likely at down channel locations.

Observational, mineralogical, and morphological data from the outflow channel also provide evidence that a secondary source of fluid may be present at site YT-N6.7m that was undetected during our sampling. This northern outflow channel has a different pigmentation in the mineral/biofilm precipitates (Figures 1, 2) than the southern outflow channel. The morphology of precipitates differs from all the other sample locations (Figure 4), and the mineral composition at the site suggests precipitates resulting from fresh fluid coming from depth, such as serpentine, calcite, and aragonite, rather than primarily weathering products, such as the montmorillonite that dominates the other outflow channel locations. Site YT-N6.7m occurs at a break in the slope, possibly due to a fracture in the bedrock, which could allow more fluid to leak from the subsurface. This location, with visible and extensive microterracettes on the vertical face of the break in slope would be ideal for investigating preservation of biofilms/minerals from serpentinizing systems.

### Implications of enrichment culturing

The most successful enrichments all grew in media formulated for aerobic heterotrophy, indicating that heterotrophic processes are likely key in the down channel locations. The three genera identified in the nitrate and iron reduction enrichments, *Brevibacillus*, *Thermobacillus*, and *Anoxybacillus*, can tolerate a wide range of temperature and pH, but most grow optimally at high pH (pH 8–10) (Shida et al., 1996; Pikuta et al., 2000; Touzel et al., 2000; Goto et al., 2004; Watanabe et al., 2007). All identified genera are aerobic, with the exception of *Anoxybacillus*, which can function as a facultative aerobe (Pikuta et al., 2000). Notably, *Anoxybacillus* is also capable of nitrate reduction, which may be a key ecological function in the subsurface environment. It is not known at this time if any of these genera are present at the source of the spring, but it is unlikely that they would be present in the subsurface, with the possible exception of *Anoxybacillus*. However, rapid growth at moderately thermophilic temperatures was observed in many enrichments, indicating a potential legacy of near subsurface environmental conditions. Ongoing 16S rRNA analysis from Yanartaş will help reveal these details in the future. The nearest cultured neighbors to organisms growing in the Yanartaş enrichments are all spore forming Bacteria. While the source of the fluid fueling this ephemeral ecosystem was pH 11.95, optimal growth occurred at pH 8–9.5 in all media, which matches the pH of the fluid down channel. The production of endospores by all three genera suggests that microorganisms living in the surface environments at Yanartaş are capable of surviving harsh conditions for extended periods of time until more favorable conditions arise, at which point growth can proceed rapidly (as in Supplemental Figure 1). Spore formation, tolerance of high pH, and heterotrophy are all functions that suit microorganisms living in an ephemeral, high pH spring ecosystem.

### The Yanartaş ecosystem

The Yanartaş seep ecosystem is more accurately described as two separate ecosystems, the subsurface and surface environments, connected by a transitional ecotone, which is represented by the source of the surface seep. Thus, the seep at YT-0m is connected to the subsurface and the surface environments, and may share properties with both systems while also exhibiting characteristics that are unique to that site. This connectivity is common in many spring type ecosystems, regardless of the fluid chemistry (e.g., Meyer-Dombard et al., 2011). This and work in progress define the relationships between the subsurface-ecotone-surface systems. Where the seep orifice functions as an ecotone between two distinct ecosystems, subsurface chemistry persists in the fluids that flow down the outflow channel, allowing transitions of metabolic function along the way. Further, the genetic and taxonomic diversity of the subsurface may be imprinted on the surface ecosystems to some degree, representing a legacy of subsurface ecosystem details.

Recall from above that the fluid down-channel has measurable DOC with an isotopic composition of ~-23‰. While not measured here, it is likely that (as in other similar field locations) the source fluid contains low DIC concentrations, depleted relative to the DIC in the fluid down-channel. The isotopic composition of solids found at the source is depleted relative to those measured down-channel, and the total carbon measured down-channel is enriched relative to that in organic carbon.

Options for carbon sources at YT-0m may resemble those in the subsurface ecosystem. Etiope et al. (2011) report the presence of a myriad of carbon-bearing gases at Yanartaş seeps, although CH_4_ is the most abundant carbon-bearing gas by several orders of magnitude. Assuming some degree of homogeneity among the gas seeps, we can use these data as estimates of the gas composition emanating from YT-0m. Primarily, that CH_4_ is ~87% of the total gases and −12.51‰, while CO_2_ is 0.08% of the total gas and the isotopic composition is between −18 and −20‰. Our own data find that total carbon in the solids at YT-0m have an isotopic composition of −20‰, with the organic carbon component at −27.7‰. Thus, it seems likely that mineral precipitates at YT-0m are recording the isotopic signature of the CO_2_ found at the source gas seep. The isotopic signature of the organic carbon at YT-0m is likely highly influenced by available DOC (not measured), which is expected to be similarly depleted relative to atmospheric CO_2_. Another option for depleted carbon sources that may influence the composition of organic carbon at YT-0m is ethane. Etiope et al. (2011) found 0.2–0.4% C2 gas at Yanartaş seeps, with an isotopic composition of −22 to −26‰ depending on sample location.

The availability of carbon sources varies with increasing distance from the transition ecotone at the orifice of the seep. We expect increased opportunity for entrainment of surface-affiliated organic carbon down channel, and increased influence of atmospheric carbon. Our data support this hypothesis. The isotopic composition of total carbon in solids collected down channel is between −11 and −12‰, and DIC measured at YT-S8.8m is −11.8‰, suggesting strongly that the majority of the solids collected down channel are composed of carbonates influenced by atmospheric carbon—precipitation of calcite given the limited DIC in the source fluids necessitates incorporation of atmospheric CO_2_ down channel. Note that the isotopic composition of DIC at YT-S8.8m is enriched by ~8‰ relative to the presumed gas composition at the source. Organic carbon in samples collected down channel is slightly enriched relative to that at the source (−25‰ compared to −27.7‰), suggesting that some component of carbon fixation may contribute to the biomass signature down channel.

While the previous work of Etiope et al. (2011) identified ~2% N_2_ in Yanartaş gases it does not appear that nitrogen fixation is a prominent ecological function of the source fluid seep as sampled at the surface. The isotopic composition of nitrogen is ~3‰ in biomass at the five Yanartaş sample locations. Nitrogen fixation tends to imprint the signature of atmospheric N_2_ on the resulting biomass, and thus we would expect that the isotopic signatures in collected biomass would be closer to 0‰ or slightly negative values as has been shown in other extreme ecosystems (Havig et al., 2011; Loiacono et al., 2012). The NH^+^_4_ concentrations at YT-0m were measureable, although low—it is possible that there is enough fixed nitrogen available at the source that subsurface communities may not need to fix nitrogen, and it is also possible that ecosystem function at depth differs from that of the related surface communities. Nitrogenase genes were detected by PCR in the solids collected from sites YT-N3.6m and YT-N6.7m—however it is possible that these genes are not active in the populations at the time of sampling. Our data instead suggest that denitrification or even nitrate reduction are the primary nitrogen cycle processes occurring both at the seep source and down channel. Enrichment cultures targeting nitrate reduction produced strains related to known nitrate reducing Bacteria (Section Implications of Enrichment Culturing). Nitrate and nitrite reductase genes are present in these enrichments, and although their activity has not been shown, the genetic capacity for nitrate/nitrite reduction and/or the first two steps of denitrification are present. Both *narG* and *nirS* genes were detected by PCR at all sample locations. Further, positive nitrogen isotope values such as those found in the solids collected at all locations at Yanartaş are expected for nitrogen that has been recycled in the ecosystem.

## Concluding remarks

Currently, descriptions of habitats in active terrestrial serpentinizing seeps include ~ a dozen locations across the globe. These sites include fluid seeps that are accessible at the surface, which provide an opportunity to study the communication between the subsurface and surface ecosystems. The small fluid seep discovered at Yanartaş represents a system that is ephemerally active over a long enough time period to preserve evidence of past microbiological activity. The fluids emanating from the gas seep resemble some of those reported at The Tablelands locale (Szponar et al., 2013), and feature a Ca^+2^-OH^−^ type solution at the source typical of actively serpentinizing systems.

Isotopic composition of carbon in fluids and solids, and of nitrogen in solids allows interpretation of carbon and nitrogen cycle functions as the deep subsurface fluids emerge and transition to the surface environment. Our data suggest that at the source of the seep, the transitional environment, biomass is recording the influence of the carbon isotopic signature of organic carbon input into the system, or perhaps ethane gas, but likely not methane gas. Down channel, however, our data point to the potential for carbon fixation in addition to heterotrophic activity. A combination of nitrogen isotope composition in solids, and PCR-based surveys of genes important in nitrogen cycling, lead to the conclusion that nitrogen fixation may not be a prominent process in the Yanartaş subsurface and surface ecosystems, but rather denitrification, nitrate reduction, or nitrification may dominate. Microorganisms cultured from downstream locations exemplify the capacity of surface ecosystems to adapt to subsurface chemistry, and potentially represent a legacy of subsurface genetic and metabolic diversity. Collectively, these data help define the unique ecosystems at Yanartaş and allow insight concerning the related subsurface ecosystem. Work in progress will identify the taxonomic, metabolic, and genomic diversity of all five locations described herein.

### Conflict of interest statement

The Reviewer, Matthew Schrenk, declares that, despite having published with author, Dawn Cardace, the review process was handled objectively and no conflict of interest exists. The authors declare that the research was conducted in the absence of any commercial or financial relationships that could be construed as a potential conflict of interest.

## Supplementary material

Supplemental Figure 1 **Typical growth in enrichments from Yanartaş sediment over a 4 h period**. Scale bar at lower left of each image is 10 μm. Note individual cells in **(A,B)** becoming progressively more entrained in biofilm in **(C,D)**.
