# Supplementary figures and images for: High pH microbial ecosystems in a newly discovered, ephemeral, serpentinizing fluid seep at Yanartaş (Chimera), Turkey
Source: Front Microbiol. 2015 Jan 19;5:723. doi: 10.3389/fmicb.2014.00723 (PMC4298219; doi:10.3389/fmicb.2014.00723)

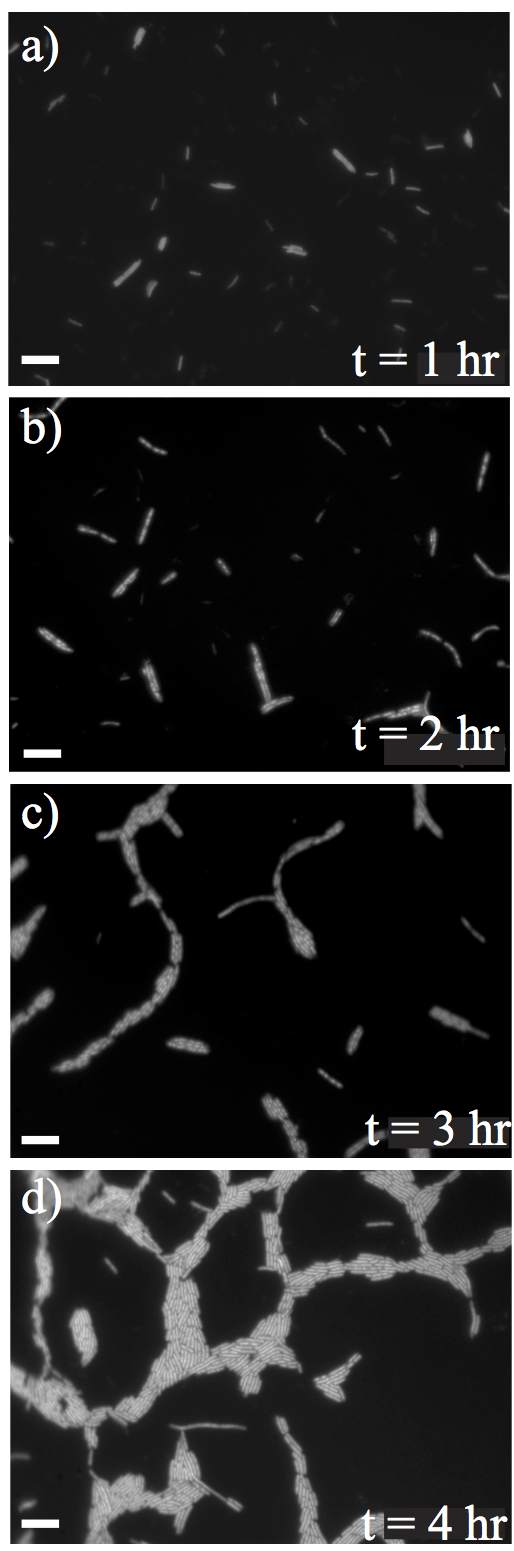

Supplement: Supplemental Figure 1 — Typical growth in enrichments from Yanartaş sediment over a 4 h period. Scale bar at lower left of each image is 10 μm. Note individual cells in (A,B) becoming progressively more entrained in biofilm in (C,D). [file Image1.TIFF]
